# Nutrients regulation of skin cells from canines and cats via Wnt/β-catenin signaling pathway

**DOI:** 10.3389/fvets.2025.1486201

**Published:** 2025-02-07

**Authors:** Xue Sun, Yan Ma, Yi Gao, Jiaxi Li, Yunliang Li, Lei Lv

**Affiliations:** ^1^Nourse Centre for Pet Nutrition, Wuhu, China; ^2^Shanghai Chowsing Pet Products Co., Ltd., Shanghai, China; ^3^Wuhu Weishi Biotechnology Co., Ltd., Wuhu, China; ^4^School of Food and Biological Engineering, Jiangsu University, Zhenjiang, China

**Keywords:** keratin hydrolyzed, egg yolk lecithin, fish collagen peptide, β-catenin, hair regeneration process

## Abstract

For pets, healthy hair not only represents their beautiful appearance, but also reflects their overall health status. Nutritional imbalance, skin parasites, and stress can all cause a large amount of hair loss in pets, leading to skin-related diseases and posing a threat to their health. So, it is very important to understand and find solutions to alleviate or treat pet hair loss. The Wnt/β-catenin signal plays a core role in the hair regeneration process. Here, we report that keratin hydrolyzed, egg yolk lecithin, and fish collagen peptide promote β-catenin nuclear translocation and significantly enhance the expression of their target genes in canines and cats skin cell lines, indicating that these nutrients are likely to play a beneficial role in promoting hair regeneration in canines and cats.

## Introduction

Pets contribute to human physical and mental health, alleviate life stress, and promote family harmony. The health of pet hair is increasingly valued by people, and the health of pet hair is crucial to their overall health and happiness. A healthy hair not only makes pets look more beautiful, but also reflects their physical condition and quality of life. Therefore, it is the responsibility of every pet owner to understand the importance of healthy pet hair. Pets such as canines and cats form a steady cycle of hair shedding and regeneration, ensuring the renewal of hair. However, many pets experience frequent hair loss due to various reasons, such as nutritional imbalance (1), skin parasites (2), and stress (3), ultimately leading to a series of skin diseases (4). So, finding effective solutions to maintain pet hair health is very important.

Wnts are a key driving factor for most types of tissue stem cells in adult mammals (5). The downstream effector protein of canonical Wnt signal transduction is β-catenin. After Wnt signal activation, β-catenin enters the nucleus, where it binds to DNA-bound TCF (T cell factor) transcription factors and affects gene transcription (6, 7). In hair follicles, Wnt signaling plays multiple roles in the biology of stem cells and progenitor cells (8). By overexpressing Dickkopf (DKK, Wnt inhibitor) to block Wnt signaling, hair follicles and other skin appendages can be eliminated (9). The activation of Wnt signaling by β-catenin can lead to the expansion of stem cells in the hair follicle system (10). In recent years, significant progress has been made in understanding the molecular basis of hair loss and identifying effective intracellular targets to design effective hair loss treatment methods (11). Although multiple growth factors and signaling pathways are involved in hair regeneration, the activation of the Wnt/β-catenin signaling pathway plays a core role in hair regeneration (12).

Keratin hydrolyzed (13), egg yolk lecithin (14), cysteine (15), fish collagen peptide (16), and cow bone collagen protein are commonly used cosmetic and health-promoting ingredients aimed at enhancing skin and hair health. However, it remains unclear whether these nutrients have an impact on hair growth in canines and cats. Therefore, we conducted studies using a canines and cats skin cell model to investigate their effects. We found that several nutrients, including hydrolyzed keratin, egg yolk phospholipids, and fish collagen peptides, enhance the localization of β-catenin in the nucleus and promote the expression of its target genes, which are crucial for the proliferation of skin cell lines and hair regeneration in canines and cats. This indicates that keratin hydrolyzed, egg yolk lecithin, and fish collagen peptide are likely to promote hair regeneration in canines and cats.

## Materials and methods

### Cell culture

Canines skin cell lines PETCC167 and cats skin cell lines PETCC172 were purchased from the PETCC (Nourse, Wuhu, China) and were cultured in DMEM medium containing FBS (10%) and penicillin/streptomycin (P/S, 1%). PETCC 167 is a canine skin tumor cell collected by PETCC in 2023, it is extracted from a 10-year-old female Toy Poodle. PETCC 172 is a feline skin tumor cell collected by PETCC in 2023, it is extracted from an 8-year-old male American Shorthair. All cells were cultured in a 37°C constant temperature incubator containing 5% CO_2_.

### Cell proliferation assay

Cells were inoculated into 96-well plates with a density of 2,000 cells per well. Subsequently, cells were treated with keratin hydrolyzed (2 mg/mL), egg yolk lecithin (0.25 mM), cysteine (1 mM), fish collagen peptide (5 mg/mL), and cow bone collagen protein (5 mg/mL) for 24 h, 48 h or 72 h, and the OD value was measured at each time point. After incubation with CCK8 reagent (Meilunbio, MA0218) at 37°C for 2 h, the absorbance at 450 nm was measured with Multiscan Spectrum (Thermo) every day to detect cell proliferation.

### Western blot

Cell lysate proteins were collected and loaded on SDS polyacrylamide gels as previously described (17). Membranes were incubated with appropriate antibodies against β-catenin (51067-2-AP, Proteintech, 1:2000), β-Actin (66009-1-Ig, Proteintech, 1:20000), Tublin (14555-1-AP, Proteintech, 1:1500) or H3 (17168-1-AP, Proteintech, 1:8000) at 4°C overnight. After washes with PBST saline membranes were incubated with a horseradish peroxidase–linked secondary antibody (1:5000) for 1 h. Immunoreactive bands were detected with an enhanced chemiluminescent plus reagent kit.

### Quantitative real-time PCR

RNA purification kit (EZBioscience) and Reverse Transcription Master Mix (EZBioscience) were used to extract total RNA from cells and perform reverse transcription reactions, respectively. Perform qRT-PCR experiment using Applied Biosystem 7,300 plus Sequence Detection System (Applied Biosystems). The endogenous controls for mRNA was β-actin RNA. The relative quantification (2^−ΔΔCT^) method was used for results analyzing. Primers were shown in Supplementary Table S1. The qPCR reaction procedure was shown in Supplementary Table S2.

### Separation of nuclear and cytoplasmic proteins

Separate the nucleus and cytoplasm of cells using the nuclear protein and cytoplasmic protein extraction kit (Beyotime, P0027), and prepare whole cell lysate for WB. All operations are carried out according to the instructions.

### Statistical analysis

The experimental data represent the mean ± standard error of three repeated experiments. Statistical software GraphPad prism 9.0 was used for statistical analysis. Comparisons between two groups for difference analysis were performed with two-tailed Student *t*-test, significance level were indicated as *p < 0.05; **p < 0.01; ***p < 0.001; ****p < 0.0001; ns, no significance.

## Results

### Keratin hydrolyzed, egg yolk lecithin, cysteine, and fish collagen peptide promote the proliferation of skin cell lines in canines and cats

**Figure 1:**
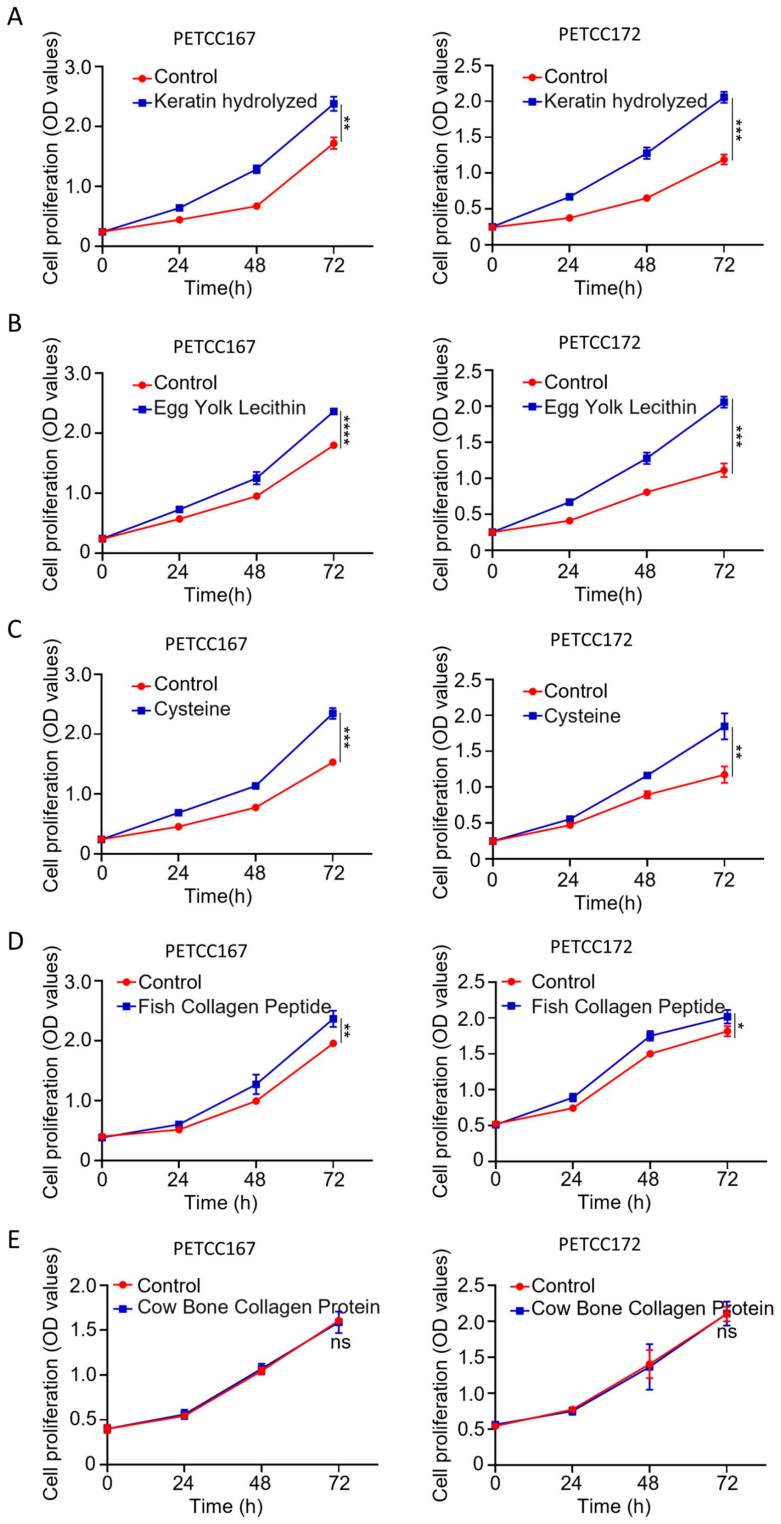
The effects of keratin hydrolyzed, egg yolk lecithin, cysteine, fish collagen peptide, and cow bone collagen protein on the proliferation of skin cells in canines and cats. Canine and cat skin cell lines were treated with keratin hydrolyzed (**A**, 2 mg/mL), egg yolk lecithin (**B**, 0.25 mM), cysteine (**C**, 1 mM), fish collagen peptide (**D**, 5 mg/mL), and cow bone collagen protein (**E**, 5 mg/mL) for 24, 48, or 72 h, and the OD value was measured at each time point. Values are means ± SD from *n* = 3 independent experiments. Statistical differences were determined by Student’s *t*-test. *p < 0.05, **p < 0.01, ***p < 0.001, ****p < 0.0001; ns, no significance.

Maintaining the proliferation ability of skin cell lines is a fundamental condition for hair regeneration. To investigate the effects of several nutrients on the proliferation of skin cell lines in canines and cats, we selected five nutrients including keratin hydrolyzed, egg yolk lecithin, cysteine, fish collagen peptide, and cow bone collagen protein. The results showed that keratin hydrolyzed (Figure 1A), egg yolk lecithin (Figure 1B), cysteine (Figure 1C), and fish collagen peptide (Figure 1D) significantly promoted the proliferation of skin cell lines in canines and cats, while cow bone collagen protein did not affect cell proliferation (Figure 1E). In summary, keratin hydrolyzed, egg yolk lecithin, cysteine and fish collagen peptide are beneficial for maintaining the proliferation ability of skin cell lines in canines and cats.

### Keratin hydrolyzed, egg yolk lecithin, and fish collagen peptide upregulate protein levels of β-catenin in canines and cats skin cell lines

**Figure 2:**
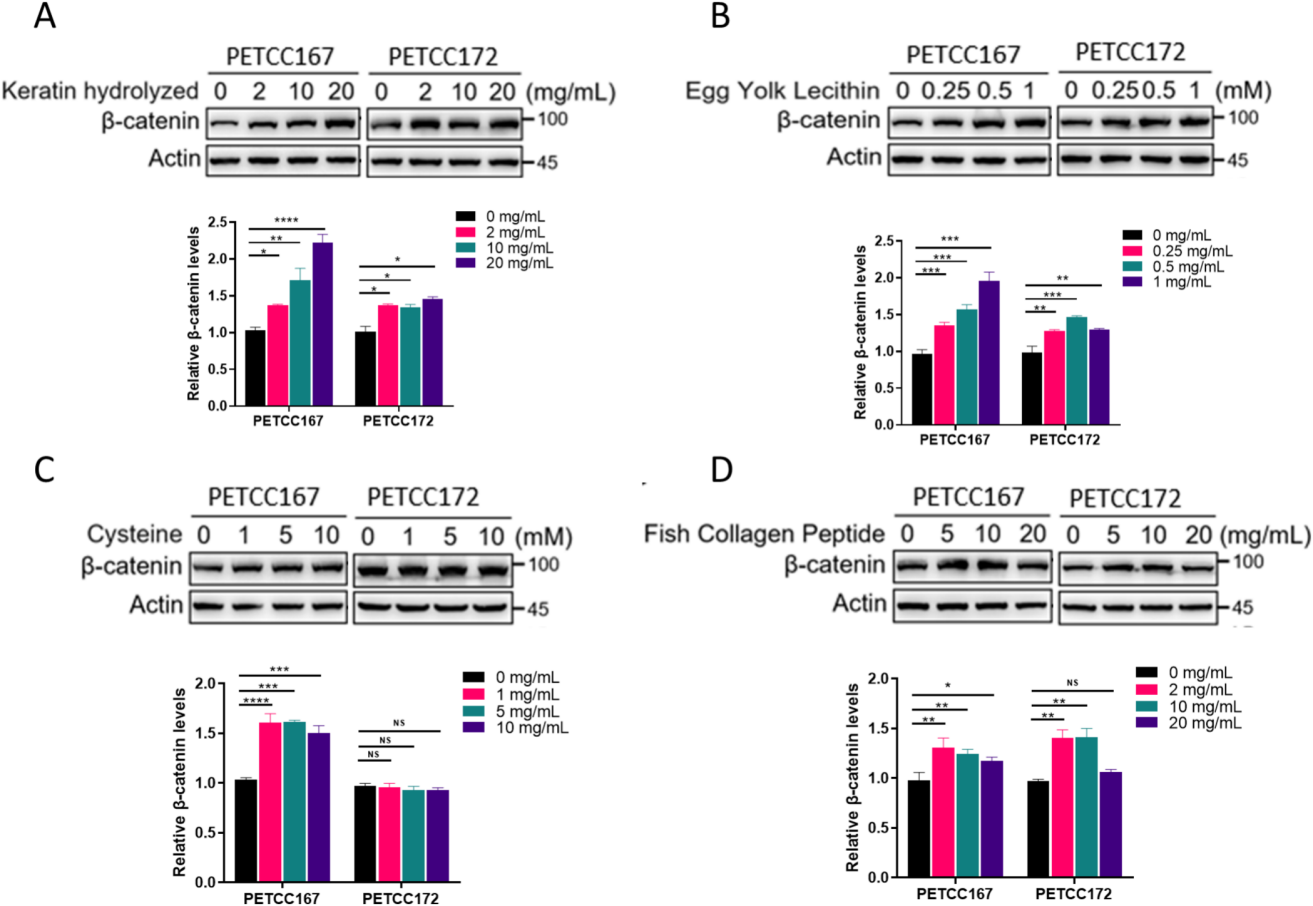
The effects of keratin hydrolyzed, egg yolk lecithin, cysteine, and fish collagen peptide on β-catenin protein levels in canine and cat skin cells. Western blot analysis of β-catenin protein level in canine and cat skin cell lines treated with or without hydrolyzed keratin **(A)**, egg yolk phospholipids **(B)**, cysteine **(C)**, and fish collagen peptides **(D)** as indicated for 48 h. The bar graph below represents the statistical analysis of β-catenin band density. Statistical differences were determined by Student’s *t*-test. *p < 0.05, **p < 0.01, ***p < 0.001, ****p < 0.0001; ns, no significance.

Given the crucial role of the Wnt/β-catenin signaling pathway in the proliferation and hair regeneration of skin cell lines, we next examined the effects of several nutrients on the β-catenin protein levels in canines and cats skin cell lines. The results showed that keratin hydrolyzed (Figure 2A), egg yolk lecithin (Figure 2B), and fish collagen peptide (Figure 2D) significantly upregulated the protein levels of β-catenin in the canines and cats skin cell lines. However, cysteine selectively affects the protein levels of β-catenin in canines skin cell line, while exhibiting no impact on skin cell line (Figure 2C). Although our results in Figure 1 have shown that cysteine promotes the proliferation of skin cell lines in canines and cats, this effect is not achieved by promoting β-catenin expression. In summary, keratin hydrolyzed, egg yolk lecithin and fish collagen peptide upregulated the protein levels of β-catenin.

### Keratin hydrolyzed, egg yolk lecithin, and fish collagen peptide promote β-catenin nuclear translocation

**Figure 3:**
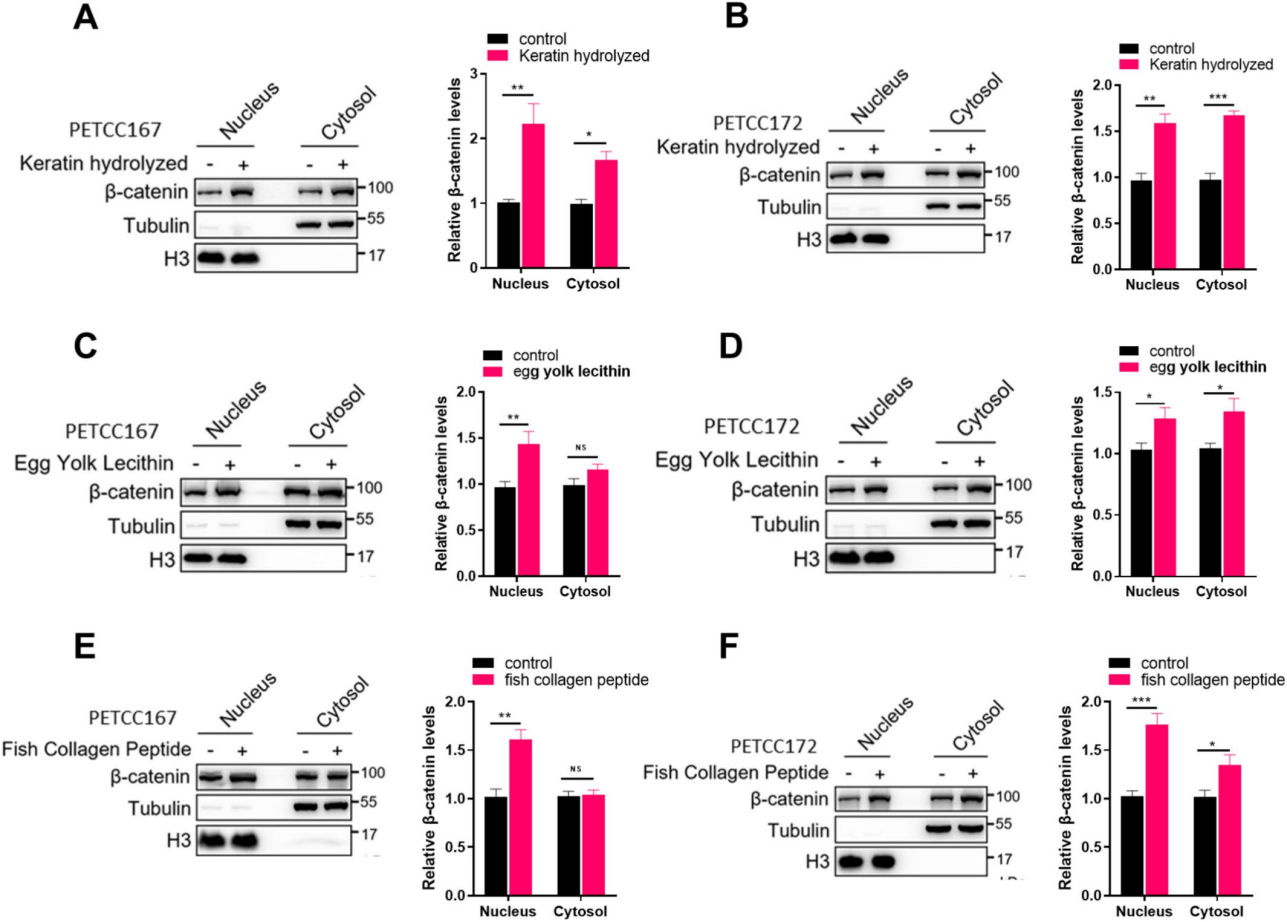
Keratin hydrolyzed, egg yolk lecithin, and fish collagen peptide promote the nuclear localization of β-catenin in canine and cat skin cells. Western blot analysis of the cytosolic and nuclear location of β-catenin in canine and cat skin cell lines treated with keratin hydrolyzed (**A,B**, 2 mg/mL), egg yolk phospholipids (**C,D**, 0.25 mM), and fish collagen protein (**E,F**, 5 mg/mL). The bar graph on the right represents the statistical analysis of β-catenin band density. Statistical differences were determined by Student’s *t-*test. *p < 0.05, **p < 0.01, ***p < 0.001; ns, no significance.

When the Wnt/β-catenin signal is activated, the β-catenin protein enters the nucleus to promote the expression of target genes, which helps with hair regeneration in hair follicles (11, 18). Our results have demonstrated that keratin hydrolyzed, egg yolk lecithin, and fish collagen peptide upregulate the protein level of β-catenin, but it is unclear whether these nutrients can promote the protein level of β-catenin in the nucleus. Next, we examined the localization of β-catenin in the nucleus of skin cell lines from canines and cats treated with keratin hydrolyzed, egg yolk lecithin, and fish collagen peptide. Our results demonstrated that keratin hydrolysate treatment enhanced β-catenin expression in both nuclear and cytoplasmic compartments of PETCC167 and PETCC172 cells (Figures 3A,B). In PETCC167 cells, egg yolk lecithin specifically increased nuclear β-catenin expression without affecting its cytoplasmic levels (Figure 3C). However, in PETCC172 cells, egg yolk lecithin treatment led to elevated β-catenin expression in both nuclear and cytoplasmic fractions (Figure 3D). Similarly, fish collagen peptide selectively enhanced nuclear β-catenin expression in PETCC167 cells (Figure 3E), while in PETCC172 cells, it increased β-catenin levels in both cellular compartments (Figure 3F). Collectively, these findings suggest that keratin hydrolysate, egg yolk lecithin, and fish collagen peptide promote β-catenin nuclear translocation.

### Keratin hydrolyzed, egg yolk lecithin, and fish collagen peptide promote the expression of β-catenin target genes

**Figure 4:**
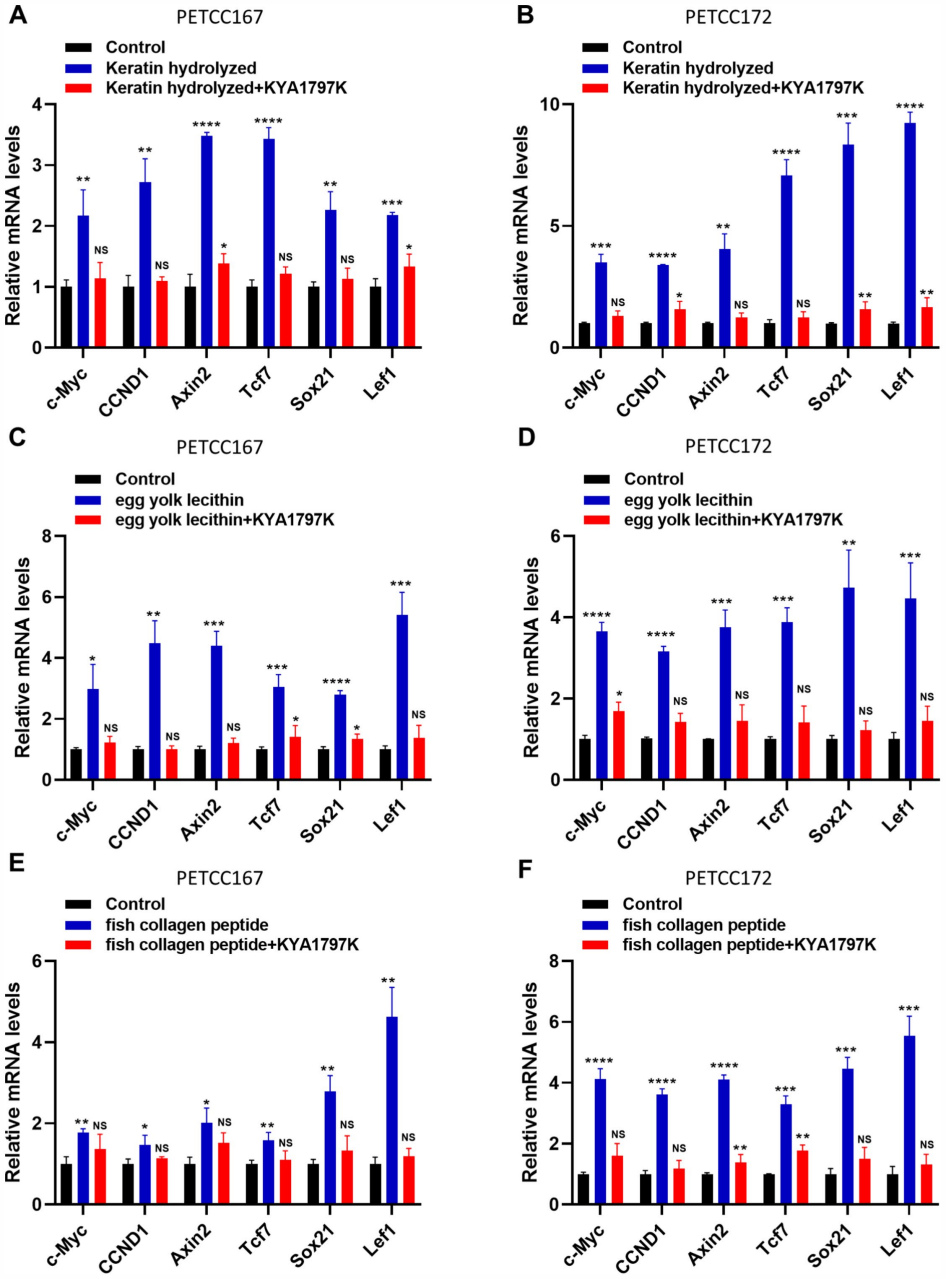
Keratin hydrolyzed, egg yolk lecithin, and fish collagen peptide promote the expression of β-catenin target genes in canine and cat skin cells. Canine and cat skin cell lines were treated with keratin hydrolyzed (**A,B**, 2 mg/mL), egg yolk phospholipids (**C,D**, 0.25 mM), fish collagen protein (**E,F**, 5 mg/mL) and KYA1797K (25 μM, Wnt/β-catenin inhibitor) for 24 h and subjected to qRT-PCR. Values are means ± SD from *n* = 3 independent experiments. Statistical differences were determined by Student’s *t*-test. *p < 0.05, **p < 0.01, ***p < 0.001, ****p < 0.0001. ns, no significance.

When β-catenin enters the nucleus, it causes the expression of target genes (19, 20). Our results have shown that keratin hydrolyzed, egg yolk lecithin, and fish collagen peptide significantly promote the nuclear translocation of β-catenin, but it is unclear whether this leads to an increase in its target gene expression. Subsequently, we examined the expression levels of target genes of β-catenin, including *c-Myc*, *CCND1*, *Axin2*, *Tcf7*, *Sox21*, and Lef1 in canines and cats skin cell lines treated with keratin hydrolyzed, egg yolk lecithin, and fish collagen peptide. Compared to the control group, keratin hydrolysate treatment significantly upregulated β-catenin target genes in both PETCC167 and PETCC172 cells, with Axin2 and Tcf7 showing the most pronounced elevation in PETCC167 cells, while Sox21 and Lef1 exhibited the highest upregulation in PETCC172 cells (Figures 4A,B). Similarly, egg yolk lecithin treatment demonstrated comparable trends in both cell lines, with Lef7 displaying the most significant upregulation in PETCC172 cells (Figures 4C,D). Consistent with these observations, fish collagen peptide treatment also enhanced the expression of these target genes, particularly Lef7 in PETCC167 cells, while Sox21 and Lef1 showed the most substantial increases in PETCC172 cells (Figures 4E,F). Furthermore, treatment with Wnt/β-catenin inhibitor KYA1797K partially reversed the upregulation of target genes induced by nutrients to varying extents, indicating that nutrients enhance gene expression levels through the Wnt/β-catenin signaling pathway (Figures 4A–F). It is worth noting that the upregulation of *c-Myc* and *CCND1* expression is consistent with the promotion of cell proliferation. More importantly, the *Axin2*, *Tcf7*, *Sox21*, and *Lef1* are crucial for hair follicle regeneration, indicating that keratin hydrolyzed, egg yolk lecithin, and fish collagen peptide may play a role in hair follicle regeneration in canines and cats.

## Discussion

The most extensively studied aspect of Wnt is its role in healthy stem cells and cancer. The effector protein downstream of canonical Wnt signaling is β-catenin, which can serve as a bipartite transcription factor for lymphoid enhancer-binding factor 1 (LEF1) and/or T-cell factor (TCF) DNA-binding proteins (21). Wnts have various effects on target cells during development. The Wnt signal can stimulate the maintenance of stem cells in hematopoietic stem cells (22) and embryonic stem cells (23) (ESCs) cultures. In these stem cells, the function of Wnt-β-catenin and LEF1-TCF has a synergistic effect. In hair follicles, Wnt/β-catenin signaling plays multiple roles in the biology of stem cells and progenitor cells (8, 24). The Wnt/β-catenin signal is the initial signal of hair follicle growth, participating in various stages of morphogenesis and cyclic cycling. It plays a key regulatory role in processes such as hair follicle placode development, papillary function, periodic changes in hair follicles, and proliferation and differentiation of skin cells (11). β-catenin is a molecular switch for Wnt signaling, cascading and integrating signals from other pathways, and is a core link in the Wnt signaling transduction pathway. Given the crucial role of the Wnt/β-catenin signaling pathway in hair follicle development and hair regeneration, targeting the Wnt/β-catenin pathway for the treatment of hair loss has become a new strategy. Here, we found that keratin hydrolyzed, egg yolk lecithin, and fish collagen peptide can significantly enhance the protein levels of β-catenin, promote its localization in the nucleus, and further upregulate the expression of its target genes, which are closely related to skin cells proliferation and hair regeneration. This indicates that feeding canines and cats food containing keratin hydrolyzed, egg yolk lecithin, and fish collagen peptide is likely to promote their hair regeneration.

β-catenin not only plays a role in the Wnt signaling pathway, but also serves as an important binding partner for the cytoplasmic tail of various cadherins (25). Cadherins and β-catenin play crucial roles in hair follicle growth and development through their involvement in cell–cell adhesion and signaling pathways. β-catenin also binds to the intracellular domain of cadherins, contributing to the stabilization of adherens junctions. In *Caenorhabditis elegans*, the two functions of β-catenin are, respectively, undertaken by two different b-catenin homologs (26). Although our research has found that keratin hydrolyzed, egg yolk lecithin, and fish collagen peptide have regulatory effects on β-catenin, the role of these nutrients in β-catenin in cadherins is still unknown and requires further exploration.

Despite significant efforts by researchers to develop safe and effective treatments for hair loss, their clinical application is limited. The Wnt/β-catenin signaling pathway plays a crucial role in stimulating skin cells and hair regeneration. Therefore, researchers have developed methods for treating hair loss by targeting this signaling pathway. Keratin hydrolyzed provide essential amino acids to canines and cats skin cells, promoting the synthesis of structural proteins and potentially enhancing the sensitivity of canines and cats skin cells to Wnt/β-catenin signaling, thereby stimulating stem cell activity. Egg yolk lecithin may improve the construction and function of cell membranes, facilitating signal transduction in canines and cats skin cells. Additionally, lipid-derived signaling molecules synthesized from these components could activate Wnt/β-catenin signaling, enhancing intercellular communication and supporting the growth and regenerative potential of hair follicles. Fish collagen peptide may directly interact with cell surface receptors or binding motifs, augmenting the transmission of Wnt/β-catenin signaling. These mechanisms warrant further investigation. But in addition to the Wnt/β-catenin signaling pathway, there are also other molecular targets, such as growth factors, notch, Gli, STAT5, etc., which are also targets for designing drugs for treating hair loss (27, 28). It is not yet known whether keratin hydrolyzed, egg yolk lecithin, and fish collagen peptide play a role in other targets besides the Wnt/β-catenin signaling pathway.

In general, our research shows that keratin hydrolyzed, egg yolk lecithin, and fish collagen peptide can regulate the protein level of β-catenin and promote the expression of its target gene in canines and cats skin cell lines, which indicates that these nutrients may play an active role in promoting hair regeneration of canines and cats, but this still needs further *in vivo* experiments to verify.

## Data Availability

The original contributions presented in the study are included in the article/Supplementary material, further inquiries can be directed to the corresponding authors.

## References

[1] Steinmetz HW Kaumanns W Dix I Heistermann M Fox M Kaup FJ. Coat condition, housing condition and measurement of faecal cortisol metabolites--a non-invasive study about alopecia in captive rhesus macaques (*Macaca mulatta*). J Med Primatol. (2006) 35:3–11. doi: 10.1111/j.1600-0684.2005.00141.x, PMID: 16430489

[2] Baneth G Nachum-Biala Y Adamsky O Gunther I. *Leishmania tropica* and *Leishmania infantum* infection in dogs and cats in Central Israel. Parasit Vectors. (2022) 15:147. doi: 10.1186/s13071-022-05272-0, PMID: 35534906 PMC9087926

[3] Brunner MAT Rufenacht S Bauer A Erpel S Buchs N Braga-Lagache S. Bald thigh syndrome in sighthounds-revisiting the cause of a well-known disease. PLoS One. (2019) 14:e0212645. doi: 10.1371/journal.pone.0212645, PMID: 30794648 PMC6386255

[4] Jordan TJM Bizikova P. Canine and feline pemphigus Foliaceus-an update on pathogenesis and treatment. Vet Clin North Am Small Anim Pract. (2024). doi: 10.1016/j.cvsm.2024.11.010 39725576

[5] Nusse R Clevers H. Wnt/beta-catenin signaling, disease, and emerging therapeutic modalities. Cell. (2017) 169:985–99. doi: 10.1016/j.cell.2017.05.016, PMID: 28575679

[6] Behrens J von Kries JP Kuhl M Bruhn L Wedlich D Grosschedl R. Functional interaction of beta-catenin with the transcription factor LEF-1. Nature. (1996) 382:638–42. doi: 10.1038/382638a0 8757136

[7] Molenaar M van de Wetering M Oosterwegel M Peterson-Maduro J Godsave S Korinek V. XTcf-3 transcription factor mediates beta-catenin-induced axis formation in Xenopus embryos. Cell. (1996) 86:391–9. doi: 10.1016/S0092-8674(00)80112-9, PMID: 8756721

[8] DasGupta R Fuchs E. Multiple roles for activated LEF/TCF transcription complexes during hair follicle development and differentiation. Development. (1999) 126:4557–68. doi: 10.1242/dev.126.20.4557, PMID: 10498690

[9] Andl T Reddy ST Gaddapara T Millar SE. WNT signals are required for the initiation of hair follicle development. Dev Cell. (2002) 2:643–53. doi: 10.1016/S1534-5807(02)00167-3, PMID: 12015971

[10] Gat U DasGupta R Degenstein L Fuchs E. De novo hair follicle morphogenesis and hair tumors in mice expressing a truncated beta-catenin in skin. Cell. (1998) 95:605–14. doi: 10.1016/S0092-8674(00)81631-1, PMID: 9845363

[11] Choi BY. Targeting Wnt/beta-catenin pathway for developing therapies for hair loss. Int J Mol Sci. (2020) 21:4915. doi: 10.3390/ijms21144915, PMID: 32664659 PMC7404278

[12] Clevers H Nusse R. Wnt/beta-catenin signaling and disease. Cell. (2012) 149:1192–205. doi: 10.1016/j.cell.2012.05.012, PMID: 22682243

[13] Villa AL Aragao MR Dos Santos EP Mazotto AM Zingali RB de Souza EP. Feather keratin hydrolysates obtained from microbial keratinases: effect on hair fiber. BMC Biotechnol. (2013) 13:15. doi: 10.1186/1472-6750-13-15, PMID: 23414102 PMC3621039

[14] Ogiso T Yamaguchi T Iwaki M Tanino T Miyake Y. Effect of positively and negatively charged liposomes on skin permeation of drugs. J Drug Target. (2001) 9:49–59. doi: 10.3109/10611860108995632, PMID: 11378523

[15] Xu W Xie B Wei D Song X. Dissecting hair breakage in alopecia areata: the central role of dysregulated cysteine homeostasis. Amino Acids. (2024) 56:36. doi: 10.1007/s00726-024-03395-5, PMID: 38772922 PMC11108903

[16] Hwang SB Park HJ Lee BH. Hair-growth-promoting effects of the fish collagen peptide in human dermal papilla cells and C57BL/6 mice modulating Wnt/beta-catenin and BMP signaling pathways. Int J Mol Sci. (2022) 23:1904. doi: 10.3390/ijms231911904, PMID: 36233206 PMC9569759

[17] Sun X Dai Y He J Li H Yang X Dong W. D-mannose induces TFE3-dependent lysosomal degradation of EGFR and inhibits the progression of NSCLC. Oncogene. (2023) 42:3503–13. doi: 10.1038/s41388-023-02856-7, PMID: 37845392

[18] Ryu YC Lee DH Shim J Park J Kim YR Choi S. KY19382, a novel activator of Wnt/beta-catenin signalling, promotes hair regrowth and hair follicle neogenesis. Br J Pharmacol. (2021) 178:2533–46. doi: 10.1111/bph.15438, PMID: 33751552 PMC8251890

[19] Joshi S De Angelis PM Zucknick M Schjolberg AR Andersen SN Clausen OPF. Role of the Wnt signaling pathway in keratoacanthoma. Cancer Rep. (2020) 3:e1219. doi: 10.1002/cnr2.1219, PMID: 32672002 PMC7941577

[20] Sun C Hu SH Dong BQ Jiang S Miao F Lei TC. Metformin promotes the hair-inductive activity of three-dimensional aggregates of epidermal and dermal cells self-assembled in vitro. Skin Pharmacol Physiol. (2022) 35:137–47. doi: 10.1159/000521400, PMID: 34883492

[21] Willert K Nusse R. Beta-catenin: a key mediator of Wnt signaling. Curr Opin Genet Dev. (1998) 8:95–102. doi: 10.1016/S0959-437X(98)80068-3, PMID: 9529612

[22] Willert K Brown JD Danenberg E Duncan AW Weissman IL Reya T. Wnt proteins are lipid-modified and can act as stem cell growth factors. Nature. (2003) 423:448–52. doi: 10.1038/nature01611, PMID: 12717451

[23] Sato N Meijer L Skaltsounis L Greengard P Brivanlou AH. Maintenance of pluripotency in human and mouse embryonic stem cells through activation of Wnt signaling by a pharmacological GSK-3-specific inhibitor. Nat Med. (2004) 10:55–63. doi: 10.1038/nm979, PMID: 14702635

[24] Lim X Tan SH Yu KL Lim SB Nusse R. Axin2 marks quiescent hair follicle bulge stem cells that are maintained by autocrine Wnt/beta-catenin signaling. Proc Natl Acad Sci USA. (2016) 113:E1498–505. doi: 10.1073/pnas.1601599113, PMID: 26903625 PMC4801317

[25] Peifer M McCrea PD Green KJ Wieschaus E Gumbiner BM. The vertebrate adhesive junction proteins beta-catenin and plakoglobin and the Drosophila segment polarity gene armadillo form a multigene family with similar properties. J Cell Biol. (1992) 118:681–91. doi: 10.1083/jcb.118.3.681, PMID: 1639851 PMC2289544

[26] Korswagen HC Herman MA Clevers HC. Distinct beta-catenins mediate adhesion and signalling functions in *C. elegans*. Nature. (2000) 406:527–32. doi: 10.1038/35020099, PMID: 10952315

[27] Legrand JMD Roy E Ellis JJ Francois M Brooks AJ Khosrotehrani K. STAT5 activation in the dermal papilla is important for hair follicle growth phase induction. J Invest Dermatol. (2016) 136:1781–91. doi: 10.1016/j.jid.2016.04.014, PMID: 27131881

[28] Gentile P Garcovich S. Advances in regenerative stem cell therapy in androgenic alopecia and hair loss: Wnt pathway, growth-factor, and mesenchymal stem cell signaling impact analysis on cell growth and hair follicle development. Cells. (2019) 8:466. doi: 10.3390/cells8050466, PMID: 31100937 PMC6562814

