# Supplementary material for: Nutrients regulation of skin cells from canines and cats via Wnt/β-catenin signaling pathway
Source: Front Vet Sci. 2025 Feb 7;12:1486201. doi: 10.3389/fvets.2025.1486201 (PMC11843729; doi:10.3389/fvets.2025.1486201)
Supplement: Supplementary file 1 [file Table_1.doc]

**Table S1: Primer sequences used in this study**

| Name | Usage | Sequence (5'-3') |
| --- | --- | --- |
| *cannie* *β-actin* | forward | TGGCACCACACCTTCTACAA |
|  | reverse | CCAGAGGCGTACAGGGATAG |
| *cannie c-Myc* | forward | CCCTCCACCAGGAAGGACTA |
|  | reverse | CGTTGTGTGTTCGCCTCTTG |
| *cannie CCND1* | forward | AGTGTGATGCGGACTGTCTC |
|  | reverse | CGCACCCTCAAATGTTCACG |
| *cannie Axin2* | forward | CCAAGTGTCTCTACCTCATT |
|  | reverse | CTCCTTCTCTTCATCCTCTC |
| *cannie Tcf7* | forward | AAGACACAGGCAGAATCC |
|  | reverse | TGGTTGATAGCAGCACTC |
| *cannie Sox21* | forward | GCACAACTCGGAGATCAG |
|  | reverse | CTTCTTGAGCAGCGTCTT |
| *cannie Lef1* | forward | GGTGGTGTTGGACAGATTA |
|  | reverse | CTGACAGTGAGGATGGATAG |
| *cat β-actin* | forward | TGGCACCACACCTTCTACAA |
|  | reverse | CCAGAGGCGTACAGGGATAG |
| *cat c-Myc* | forward | AGCAAACCTCCTCACAGCCC |
|  | reverse | ACTGTCCAACTTGACCCTCT |
| *cat CCND1* | forward | AGTTCATTTCCAACCCGCCT |
|  | reverse | AGACAGTCCGCGTCACACTT |
| *cannie Axin2* | forward | CCAAGTGTCTCTACCTCATT |
|  | reverse | CTCCTTCTCTTCATCCTCTC |
| *cat Tcf7* | forward | TCAACCAGCAGACGGATT |
|  | reverse | ACTGTCATCGGAAGGAACT |
| *cat Sox21* | forward | GCACAACTCGGAGATCAG |
|  | reverse | CTTCTTGAGCAGCGTCTT |
| *cat Lef1* | forward | ATGAATTAGCACGGAAGGAA |
|  | reverse | ATCTGTAGGTAGGATGAAGAAG |

**Table S2: qPCR reaction procedure**

| Step | Temperature | Time | Cycle |
| --- | --- | --- | --- |
| predegeneration | 95℃ | 2min | 1 |
| predegeneration | 95℃ | 10s | 40 |
| Annealing | 60℃ | 20s (Acquisition fluorescence) |
| melting curve | 60℃-95℃ | 2℃/s (Acquisition fluorescence) | 1 |
